# Cervicofacial Surgical Emphysema Following Tonsillectomy: A Rare Complication Linked to Mucosal Disruption and Air Tracking

**DOI:** 10.1155/crot/8599934

**Published:** 2025-12-07

**Authors:** Liam D. Hyland, Chris Bodimeade, Mohamed Elmorsy, Mohammed Salem

**Affiliations:** ^1^ Queen’s Medical Centre, Nottingham University Hospitals NHS Trust, Nottingham, UK, nhs.uk; ^2^ East Surrey Hospital, Surrey and Sussex Healthcare NHS Trust, Redhill, UK, surreyandsussex.nhs.uk; ^3^ James Cook University Hospital, South Tees Hospitals NHS Foundation Trust, Middlesbrough, UK, southtees.nhs.uk; ^4^ Lincoln County Hospital, United Lincolnshire Hospitals NHS Trust, Lincoln, UK, ulh.nhs.uk

**Keywords:** cervicofacial, complication, conservative management, crepitus, material risk, mediastinum, post-tonsillectomy, surgical emphysema

## Abstract

Tonsillectomy is one of the most frequently performed procedures in ear, nose and throat (ENT) surgery, commonly indicated for recurrent tonsillitis, recurrent peritonsillar abscess, obstructive sleep apnoea or suspected malignancy. While generally safe, it carries recognised risks including haemorrhage, infection, pain and orodental trauma. Surgical emphysema is a rare but clinically significant complication, with fewer than 30 cases reported and an estimated incidence below 0.02%. It is hypothesised to result from mucosal or muscular disruption of the pharyngeal wall during dissection, particularly with electrocautery or aggressive technique, exacerbated by postoperative factors such as coughing, vomiting or Valsalva manoeuvres. We present the case of a 25‐year‐old woman who underwent elective tonsillectomy for recurrent tonsillitis. The procedure was uneventful, but she re‐presented within 24 h with left‐sided jaw and neck pain. Examination revealed cervicofacial crepitus, and CT imaging confirmed surgical emphysema extending from the submandibular region to the superior mediastinum and anterior chest wall. She was admitted for observation and treated conservatively with intravenous antibiotics and analgesia. Her symptoms resolved within 3 days, and she was discharged without further intervention. This case highlights the importance of early recognition and appropriate management of post‐tonsillectomy surgical emphysema. Although rare, its potential severity, particularly in patients with respiratory comorbidities, warrants inclusion in preoperative counselling. In line with the Montgomery ruling, clinicians should consider individual risk factors when discussing consent. Greater awareness of this complication may support timely diagnosis and reinforce the value of nuanced risk communication in ENT practice.

## 1. Introduction

Tonsillectomy is one of the most commonly performed elective procedures, especially in paediatric and adult ENT practice, the indications for which include recurrent episodes of acute tonsillitis that significantly impair daily functioning, recurrent peritonsillar abscess, obstructive sleep apnoea or sleep disordered breathing, and an asymmetrically enlarged tonsil suspicious for malignancy [1, 2]. Tonsillectomy, while generally safe, carries a recognised risk of complications, most commonly postoperative haemorrhage, pain, infection and anaesthetic‐related issues such as orodental trauma and aspiration [3]. However, the presence of rare yet potentially life‐threatening complications such as postoperative surgical emphysema warrants greater attention [3, 4].

Post‐tonsillectomy surgical emphysema is characterised by the presence of air within the tissues under the skin, typically in the neck and face, following a tonsillectomy; some reports suggest that it occurs due to inadvertent injury to the pharyngolaryngeal wall through mucosal or muscular disruption. This can occur when the tonsillar capsule is breached during dissection, especially with electrocautery or aggressive instrumentation, thus creating a potential pathway for air to enter the parapharyngeal or retropharyngeal spaces. Postoperative factors such as forceful coughing, vomiting or repeated Valsalva manoeuvres can exacerbate this by increasing oropharyngeal pressure, driving air into these fascial planes [5, 6]. A literature review from 2000–2020 identified only 26 reported cases involving this complication, equating to an estimated incidence of < 0.02% across all tonsillectomy procedures [4]. Most patients present within the first 24 h postsurgery, and despite its low occurrence, the potential severity of surgical emphysema, from airway compromise to mediastinal extension, raises significant concerns that extend beyond its statistical rarity [4, 5].

The current consenting process for tonsillectomy typically addresses common risks such as pain, bleeding, infection and orodental trauma; however, the omission of rarer but more serious complications may not fully align with evolving medicoethical standards [7, 8]. According to the Montgomery vs. Lanarkshire ruling, risks must be disclosed when they are considered material by a reasonable patient, regardless of their statistical rarity [7]. Though risks above 1% are commonly discussed during consent, even those far below that threshold warrant disclosure when the severity of harm is substantial or when individual patient concerns suggest it would be meaningful [6–8].

## 2. Case Presentation

A 25‐year‐old patient was seen in the ENT clinic for recurrent tonsillitis; she reported having suffered [7] acute episodes per year during the course of the past 2 years. Examination revealed bilateral Brodsky grade [2] unhealthy scarred tonsils; she fulfilled the Scottish Intercollegiate Guidelines Network criteria for a tonsillectomy and so was consented for this procedure. The patient had a medical background of asthma, menorrhagia, irritable bowel syndrome and inappropriate tachycardic syndrome for which she was under investigation by Cardiology. She was an ex‐smoker, occasionally consumed alcohol and worked for the ambulance service. Her only known allergy was to waterproof plasters.

The patient underwent tonsillectomy 2 months later on the elective surgical list. A right angled endotracheal (RAE) tube was easily inserted, and the tonsils were dissected via a combined cold steel and bipolar approach. She was extubated without complication and did not suffer any persistent coughing or vomiting postoperatively. The patient remained well in recovery and was discharged home 6‐h post‐op with a course of antibiotics (Co‐amoxiclav 625 mg three times a day for 7 days) and analgesia (codeine, paracetamol and ibuprofen as required).

The following day (Day 1 post‐op), she returned to the emergency department with left‐sided jaw and neck pain along with left aural fullness and pressure in her throat leading to a muffled voice. She denied any dysphagia, odynophagia or difficulty in breathing but was struggling to elicit a cough. There was no post‐tonsillectomy bleeding. On examination, she had palpable crepitus running along the angle of the left mandible and tracking down the anterior neck towards the clavicle. There were no overlying skin changes detected, and the area was not warm to the touch.

**Figure 1:**
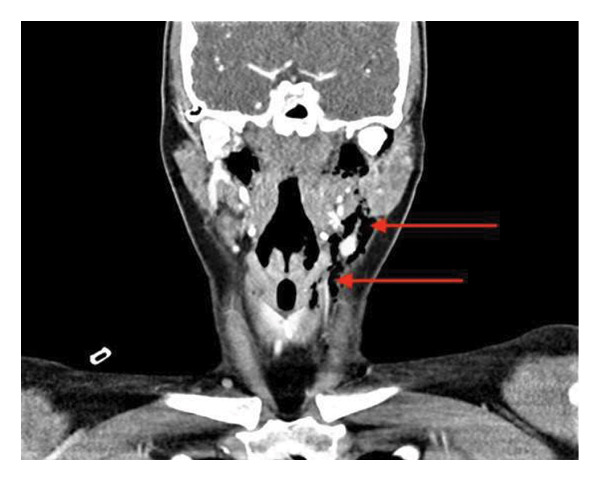
Coronal view of a CT neck, in soft tissue window, showing surgical emphysema (red arrows) situated around the ramus of the left mandible and extending inferomedially to the strap muscles and thyroid cartilage.

**Figure 2:**
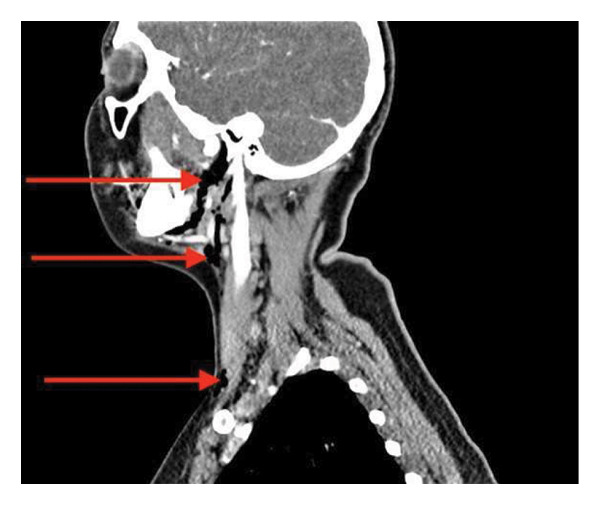
Sagittal view of a CT neck, in soft tissue window, showing surgical emphysema (red arrows) situated in the oropharynx, below the angle of the mandible and extending inferiorly to the supraclavicular space.

A flexible nasendoscopy was performed which showed mucus congestion of the nasal passages, nasopharynx and oropharynx, but no obvious tear or laceration in the tonsillar fossa. An urgent chest X‐ray was performed which showed surgical emphysema within the soft tissues of the neck on the left‐hand side; the heart and cardiomediastinal contours were normal with clear lung fields and no evidence of pneumothorax. A computed tomography (CT) scan of the neck and thorax with contrast was conducted which revealed left‐sided cervicofacial surgical emphysema originating in the left submandibular region and extending inferiorly into the superior mediastinum and left anterior chest wall (see Figures 1 and 2).

A number of differential diagnoses were considered, the most significant of which was necrotising fasciitis; the presence of crepitus and rapid spread meant that this differential was initially suspected; however, the patient’s inflammatory markers remained static (white cell Count 8 and C reactive Protein 16), and there was a lack of erythematous skin changes as would be expected in an infective process. For this reason, the differential of cellulitis was also excluded. An alternative differential was an allergic reaction causing bullous formation of the skin. Given the patient’s history of allergy to waterproof plasters, she was at an increased risk; however, neither dressings had been applied to the area nor did she exhibit any other signs of a severe allergic reaction such as pruritus or respiratory distress.

Therefore, following multidisciplinary team discussion between the ENT on‐call consultant, registrar and senior house officer as well as on‐call radiologist, the diagnostic consensus was that of surgical emphysema, and this was supported by the CT imaging.

The patient was immediately admitted under ENT, and a conservative management plan was adopted to include close observations of airway symptoms, commencement of intravenous antibiotics (co‐amoxiclav and metronidazole) and administration of regular analgesia (paracetamol, ibuprofen and benzydamine hydrochloride spray). The patient was continually observed on the ward for any signs of airway compromise as well as monitoring for further spread of surgical emphysema. The patient’s clinical state quickly improved and 3 days following readmission, she had a repeat chest X‐ray which showed complete resolution of the surgical emphysema. She was subsequently discharged and was advised to complete a 1‐week course of oral antibiotics.

A follow‐up consultation 1 week since her readmission revealed that the pain and swelling had both fully resolved. There were neither fevers, dysphagia or breathing difficulties nor any post‐tonsillectomy bleed. Her previously muffled voice had also returned to normal. The patient made a full recovery with conservative management alone. She was subsequently contacted via telephone 6 months later, which confirmed that she had returned to work and was in good health.

## 3. Discussion

Surgical emphysema is a rare complication of tonsillectomy, with fewer than 30 cases reported in the literature over the past 2 decades [4, 5]. Its pathogenesis is multifactorial, involving both anatomical vulnerabilities and physiological triggers. Proposed mechanisms include mucosal or muscular disruption of the pharyngolaryngeal wall, which allows air to dissect through the superior constrictor muscle into the parapharyngeal and retropharyngeal spaces [9, 10]. This can be exacerbated by increased upper airway pressure from coughing, vomiting or straining, facilitating air migration along fascial planes into the neck and potentially the mediastinum [11]. Alternatively, alveolar rupture due to elevated intrathoracic pressure may result in pneumomediastinum with secondary extension into cervical tissues [12]. A third, less common theory involves gas‐producing organisms or chemical reactions within enclosed tissue spaces [13].

Notably, this patient’s history of asthma may have contributed to increased intrathoracic pressure during coughing or straining episodes. Asthmatic patients are also more prone to vigorous respiratory efforts postextubation, which can exacerbate submucosal air entry [11]. Additionally, her inappropriate tachycardic syndrome may reflect underlying autonomic dysregulation, potentially amplifying physiological responses to surgical stress or airway irritation. Her status as an ex‐smoker could suggest residual airway hyperreactivity or compromised mucosal integrity, further increasing susceptibility to pressure‐related complications. Taken together, these factors may have created a physiological environment more conducive to the development of cervicofacial emphysema, despite the rarity of this complication.

The extent and distribution of surgical emphysema varies widely. Whilst most cases are confined to the neck and submandibular region, others have demonstrated spread to the chest wall, axillae and even retroperitoneal spaces [14]. Severity ranges from benign, self‐limiting crepitus to life‐threatening airway compromise and mediastinitis [15]. Diagnostic imaging, particularly CT, is essential to delineate the anatomical spread and exclude deeper complications such as a pneumothorax [16].

Management strategies are tailored to severity. Conservative treatment remains the mainstay in uncomplicated cases, comprising close observation, supplemental oxygen to promote nitrogen washout, prophylactic antibiotics to prevent secondary infection and avoidance of activities that increase airway pressure [17]. In the present case, the absence of airway compromise, haemodynamic instability, or radiological evidence of mediastinal extension, supported a conservative approach. More aggressive interventions such as tracheostomy, surgical decompression or mucosal repair are reserved for cases with progressive symptoms, acute airway compromise or respiratory distress [16, 17]. The decision to avoid invasive measures in this case was further justified by the patient’s clinical improvement and radiological resolution within days.

Given its rarity, surgical emphysema is not routinely discussed during consent for a tonsillectomy. However, its potential severity warrants consideration. While the incidence is extremely low, the consequences, particularly in cases involving airway compromise or mediastinitis, can be significant. Informed consent should reflect a balanced discussion: emphasising the overall safety profile of tonsillectomy while acknowledging rare but serious complications. This is especially pertinent in patients with anatomical risk factors, such as dense adhesions from recurrent tonsillitis or prior peritonsillar abscess [18].

Even though most cases of post‐tonsillectomy surgical emphysema resolve within 14 days, the variable extent and severity documented in the literature, from cervicofacial spread to pneumothorax, warrants heightened clinical awareness. Given the serious nature of even rare outcomes, this complication may constitute a material risk, aligning with the legal expectations outlined in Montgomery vs. Lanarkshire Health Board. As such, consideration should be given to including surgical emphysema in consent discussion, particularly in patients with anatomical or procedural risk factors.

## 4. Conclusion

This case highlights the importance of early recognition and appropriate management of post‐tonsillectomy surgical emphysema: a rare complication with potentially life‐threatening consequences such as airway compromise and mediastinal spread. In this case, conservative management with observation, oxygen, antibiotics and analgesia proved effective, and escalation to surgical intervention was avoided due to the absence of clinical deterioration. Ultimately, this case reinforces that rare complications demand not only sound clinical judgement but also ethically robust communication.

## Consent

Patient consent was granted for use of their personal information in this case presentation.

## Conflicts of Interest

The authors declare no conflicts of interest.

## Funding

The authors confirm that no funding was received for this work.

## Data Availability

The authors confirm that the data that support the findings of this study are available from the corresponding author upon reasonable request.
